# Differences in the Susceptibility of Human Tubular Epithelial Cells for Infection with Orthohantaviruses

**DOI:** 10.3390/v15081670

**Published:** 2023-07-31

**Authors:** Pamela Schreiber, Ann-Kathrin Friedrich, Gefion Gruber, Christian Nusshag, Lukas Boegelein, Sandra Essbauer, Josephine Uhrig, Martin Zeier, Ellen Krautkrämer

**Affiliations:** 1 Department of Nephrology, University of Heidelberg, D-69120 Heidelberg, Germany; 2 Bundeswehr Institute of Microbiology, Department Virology and Intracellular Agents, German Centre for Infection Research, Munich Partner Site, D-80937 Munich, Germany

**Keywords:** orthohantavirus, kidney, acute kidney injury, tubular epithelium, cell-to-cell contacts, receptor, Hantaan virus, Tula virus, pathogenesis

## Abstract

Diseases induced by infection with pathogenic orthohantaviruses are characterized by a pronounced organ-specific manifestation. Pathogenic Eurasian orthohantaviruses cause hemorrhagic fever with renal syndrome (HFRS) with often massive proteinuria. Therefore, the use of a relevant kidney cell culture would be favorable to analyze the underlying cellular mechanisms of orthohantavirus-induced acute kidney injury (AKI). We tested different human tubular epithelial cell lines for their suitability as an in vitro infection model. Permissiveness and replication kinetics of highly pathogenic Hantaan virus (HTNV) and non-/low-pathogenic Tula virus (TULV) were analyzed in tubular epithelial cell lines and compared to human primary tubular epithelial cells. Ana-lysis of the cell line HK-2 revealed the same results for viral replication, morphological and functional effects as observed for HTNV in primary cells. In contrast, the cell lines RPTEC/TERT1 and TH1 demonstrated only poor infection rates after inoculation with HTNV and are unusable as an infection model. While pathogenic HNTV infects primary tubular and HK-2 cells, non-/low-pathogenic TULV infects neither primary tubular cells nor the cell line HK-2. Our results show that permissiveness of renal cells varies between orthohantaviruses with differences in pathogenicity and that HK-2 cells demonstrate a suitable in vitro model to study viral tropism and pathogenesis of orthohantavirus-induced AKI.

## 1. Introduction

The pathogenicity of Eurasian members of the genus Orthohantavirus (family *Hantaviridae*), which cause hemorrhagic fever with renal syndrome (HFRS), varies enormously. Tula virus (TULV) is an apathogenic or low-pathogenic orthohantavirus because only very few cases with mild disease were reported [1,2]. Other members cause mild to moderate disease (e.g., Puumala virus (PUUV), Kurkino virus), whereas Hantaan virus (HTNV) and Dobrava-Belgrade virus represent highly pathogenic members with case fatality rates (CFR) of 10–12% [3]. HFRS is characterized by acute kidney injury (AKI) and often massive proteinuria [4,5]. Hantaviral genome and antigen is detected in different cell types of the human kidney. Light and electron microscopy studies of renal biopsy samples derived from patients with acute HFRS demonstrate glomerular and tubular morphological changes [6,7,8,9,10,11]. Markers of glomerular and tubular damage are also elevated in urine of HFRS patients and correlate with disease severity [11,12,13]. The predominant involvement of the kidney is specific for the pathogenic Eurasian orthohantaviruses, whereas orthohantaviruses in the Americas cause hantaviral cardio-pulmonary syndrome (HCPS) that mainly manifests in the lung. However, the viral determinants for the specific organ tropism and the underlying molecular mechanisms of organ failure induced by infection with Eurasian or American orthohantavirus are not completely understood. In addition, the number of described orthohantaviruses is growing by identification of novel members in asymptomatically, but chronically, infected animal host species [14]. Their pathogenic potential is not yet known and cannot be determined without detection of viral genome in patients.

The analysis of pathogenesis and identification of members with risk for human disease is hampered by the absence of a reliable small animal model that displays a clinical picture resembling HFRS in humans [15]. Therefore, hantaviral research strongly depends on in vitro infection studies and requires adequate in vitro cell culture models to investigate replication and to analyze viral pathogenicity.

Pathogenic Eurasian orthohantaviruses infect human glomerular and tubular cell types [10,16,17]. Infection induces several direct effects in the renal target cells: disassembling of cell-to-cell contacts, rearrangement of the actin cytoskeleton, impairment of adhesion and motility capacity, and changes in the protein expression profile and in the interferon response, but viability of cells is not affected [10,17,18]. These alterations are cell type- and virus-specific and may play a significant role in the pathogenesis of acute kidney injury in hantavirus infection. Cytoskeletal rearrangements are observed in human podocytes but not in the Vero E6 cell line, which is derived from tubular epithelial cells of the kidney of the African Green Monkey and which is often used in virological in vitro cell culture studies [19,20]. Motility and adhesion are disturbed in human podocytes, primary glomerular endothelial cells, and tubular epithelial cells but not in glomerular mesangial cells [21]. This specificity of effects underlines the importance of using relevant cell types in infection studies. The morphological and functional changes may directly influence kidney function, because the integrity of actin cytoskeleton and cell-to-cell contacts as well as motility and adhesion capacity are prerequisites for the maintenance of apico-basolateral polarity and for proper barrier function of cellular monolayers in the kidney [22,23,24].

Despite the predominant and specific involvement of the kidney in orthohantavirus infection, in vitro studies in human renal cells are still sparse. The use of primary human kidney cells is very expensive. Primary cells have a very limited lifespan, and only few passages of sub-culturing are possible before cells change their phenotypic and functional properties. Results may also be influenced by donor-specific effects. Therefore, human renal cell lines would be a valuable and cost-saving model system in orthohantaviral research. The in vitro analysis may be helpful to analyze direct effects and to identify the underlying molecular mechanisms as well as virus-specific differences between pathogenic and non-pathogenic members of orthohantaviruses. However, all renal cells are highly differentiated and specialized cell types, which exert specific functions that were often lost in cell lines during the process of immortalization [25]. In previous studies, we tested the human podocyte cell line CIHP (conditionally immortalized human podocytes) as an in vitro cell culture model of orthohantavirus infection; the comparison with primary human podocytes revealed similar results in infection and its resulting functional effects [10,18]. In contrast to human podocytes, a plethora of different immortalized cell lines of human tubular epithelial cells exists. However, their phenotypic and functional characteristics often do not reflect the attributes from cells in situ [26]. Therefore, we evaluate three different commercially available cell lines derived from human tubular epithelium for their permissiveness and suitability as cell culture model for orthohantavirus infection compared to primary tubular epithelial cells.

## 2. Materials and Methods

### 2.1. Cells and Viruses

Three immortalized cell lines derived from human renal proximal tubular epithelium were used. HK-2 (human kidney-2) cells (ATCC, Manassas, VA, USA) and TH1 cells (kerafast, Boston, MA, USA) were maintained in DMEM with 10% fetal calf serum (FCS). RPTEC/TERT1 (renal proximal tubular epithelial cell/human telomerase reverse transcriptase 1) cells (evercyte, Wien, Austria) were maintained in DMEM/F12 medium with 10% FCS. Primary human renal epithelial cells (HREpC) were purchased from Promocell (Heidelberg, Germany) and maintained in Renal Epithelial Cell Growth Medium 2 (Ready-to-use). Only HREpCs from passage 1 to 5 were used for experiments. Hantaan virus (HTNV) strain 76-118 and Tula virus (TULV) strain Moravia were propagated and titrated on Vero E6 cells. Vero E6 cells were maintained in DMEM with 10% FCS. Studies with orthohantaviruses were performed in BSL-2 (TULV) and BSL-3 (HTNV) facility.

### 2.2. Infection of Tubular Cells with Orthohantaviruses

Tubular epithelial cells were incubated with viral inoculum (HTNV or TULV) at an MOI (multiplicity of infection) of 1 for one hour. After a triple wash with medium, fresh medium was added and cells were incubated for the indicated time points. Infection was analyzed by the detection of nucleocapsid protein (N protein) via Western blot and immunofluorescence staining. The percentage of infected cells was quantified by counting cells positive for N protein staining.

### 2.3. Immunofluorescence

For immunofluorescence, cells grown on coverslips were fixed with 3% paraformaldehyde. The following primary antibodies were used for staining: mouse anti-cytokeratin 18 (CK18) (clone RGE-53, Millipore, Burlington, MA, USA), mouse anti-N protein HTNV (B5D9, Progen, Heidelberg, Germany), mouse anti-N protein PUUV (A1C5, Progen) for TULV N protein, and rabbit anti-ZO-1 (Zonula Occludens-1) (Invitrogen, Karlsruhe, Germany). Cell nuclei were stained by Hoechst 33,342 (Invitrogen, Waltham, MA, USA). Images were taken using an Axiocam 506 mono camera attached to an Axio Observer. D1 inverted microscope (Carl Zeiss, Wetzlar, Germany).

### 2.4. Western Blot Analysis

Cellular lysates and cell-free supernatants of infected and uninfected cells were analyzed by Western blot. The following primary antibodies were used: rabbit anti-HTNV N protein for the detection of HTNV N protein, rabbit anti-PUUV N protein for the detection of TULV N protein, and mouse anti-α-tubulin (Sigma, Deisenhofen, Germany) as loading control. Near infrared fluorescent dye (IRDye)-conjugated secondary antibodies and the Odyssey CLxfrared imaging system (Li-Cor, Lincoln, NE, USA) were used for detection.

### 2.5. Flow Cytometry

For flow cytometry analysis of surface expression of hantaviral receptors, tubular cells were stained with phycoerythrin (PE)-conjugated mouse anti-integrin αVβ3 antibody (clone LM609, Millipore) together with allophycocyanin (APC)-conjugated anti-CD55 (clone IA10, BD Pharmingen, Heidelberg, Germany) and control cells were stained with the corresponding fluorescently labelled isotype antibodies. Flow cytometry analysis was performed with FACSCalibur (BD Pharmingen). To exclude non-viable cells from analysis, cell viability was monitored in parallel by using Via-Probe™ Cell Viability Solution (BD Pharmingen) according to manufacturer’s instructions.

### 2.6. Viability Assay

At day six post infection, viability of uninfected and HTNV-infected HK-2 cells was determined by measuring the amount of ATP using CellTiter-Glo luminescent cell viability assay (Promega, Walldorf, Germany) according to manufacturer’s instructions.

### 2.7. Adhesion Assay

Uninfected or HTNV-infected tubular cells at day six post infection were detached by trypsin/EDTA solution. Equal volumes of cell suspensions in fresh medium were added in a 96-well microtiter plate (10,000 cells/well) and left to adhere for 30 min at 37 °C. Afterwards, cells were washed three times with phosphate buffered saline (PBS) to remove non-adherent cells. Adhered cells were fixed, stained with Sapphire700 (Li-Cor) and DRAQ5 (BioStatus, Shepshed, UK), and quantified by scanning with Odyssey CLx infrared imaging system (Li-Cor). The amount of adhered uninfected cells was set to 100%. Three independent experiments were performed.

### 2.8. Motility Assay

Motility of tubular epithelial cells was analyzed by live cell imaging and single cell tracking. Uninfected and HTNV-infected cells (10,000 cells/cm^2^) were seeded on µ-slide 2-wells (Ibidi, Gräfelfing, Germany). Motility of cells was monitored for eight hours with a JuLiSmart Fluorescence Cell Imager (Digital-Bio, Seoul, Korea). Single cells (*n* = 30) of infected and uninfected cells were tracked via the ImageJ manual tracking plugin (Ibidi). The chemotaxis tool plugin (Ibidi) was used for analysis of tracked distances. Three independent experiments were performed, and the distance covered by uninfected cells was set to 100%.

### 2.9. Statistical Analysis

Statistical analysis was performed using Prism 5.0 (Graphpad Software Inc., San Diego, CA, USA). Normal distribution of measured values was tested with the Shapiro–Wilk test. Values of two groups were compared using two-tailed Student’s *t*-test. *p* values of <0.05 were considered significant. ns: not significant; * *p* < 0.05; ** *p* < 0.005; *** *p* < 0.0001.

## 3. Results

### 3.1. Expression of Epithelial Marker Protein Cytokeratin 18 (CK18) and Hantaviral Receptors by Human Tubular Epithelial Cell Lines

**Figure 1:**
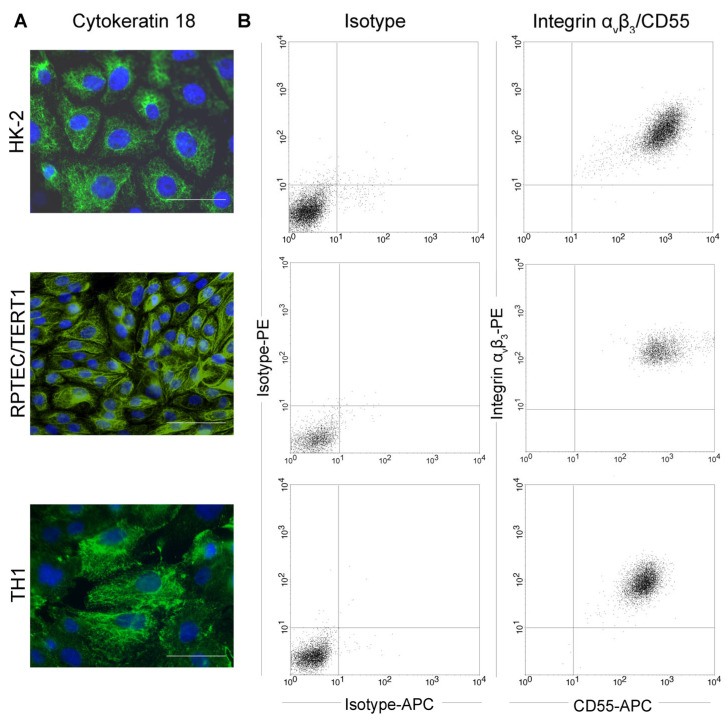
Characterization of human tubular epithelial cell lines. HK-2, TH1, and RPTEC/TERT1 cells were (**A**) stained for the epithelial marker protein cytokeratin 18 (CK18). Scale bar: 100 µm. (**B**) Surface expression of orthohantaviral entry receptors integrin αvβ3 and CD55 was detected on viable cells by flow cytometry.

We tested three different immortalized cell lines derived from human proximal tubular epithelial cells for their suitability as in vitro cell culture model of orthohantavirus infection: HK-2 cells immortalized via human papilloma virus 16 (HPV 16) E6/E7 [27], TH1 and RPTEC/TERT1 cells immortalized by human telomerase reverse transcriptase (TERT) in combination with the SV40 (Simian virus 40) T antigen or TERT alone, respectively [28,29]. First, we confirmed the epithelial origin of the three cell lines by the detection of epithelial marker protein cytokeratin CK18 via immunofluorescence and we examined surface expression of receptors integrin αvβ3 and CD55 by flow cytometry. Both receptors are described to mediate cellular entry of orthohantaviruses and are expressed on the surface of primary tubular cells [10,30,31]. The three analyzed tubular epithelial cell lines demonstrate expression of CK18 and are positive for surface expression of integrin αvβ3 together with CD55 (Figure 1).

### 3.2. Infection of Tubular Epithelial Cell Lines with HTNV

**Figure 2:**
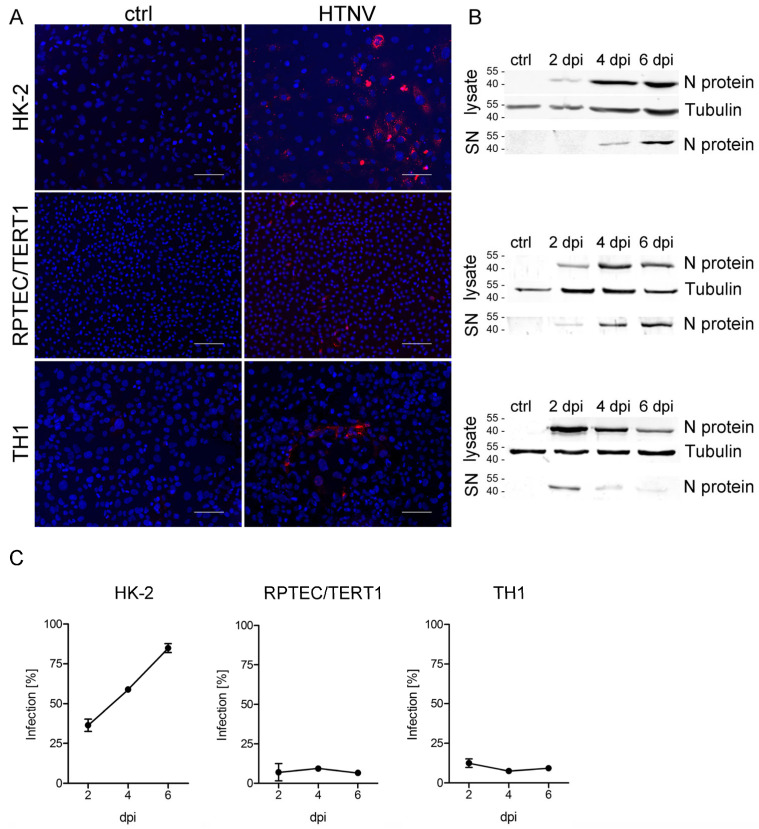
Infection of tubular epithelial cell lines with Hantaan virus (HTNV). Cells were inoculated with HTNV, and infection was monitored by detection of nucleocapsid protein (N protein) by (**A**) immunofluorescence and (**B**) Western blot analysis of cellular lysates and cell-free supernatants (SN). Shown is a representative image at six days post infection (dpi). Scale bar: 100 µm. (**C**) Percentage of infected cells was quantified by counting cells expressing N protein. Three independent experiments were performed. Mean and standard deviation (±SD) are shown.

In the next step, we analyzed the permissiveness of tubular cell lines for infection with highly pathogenic HTNV. HTNV was demonstrated to infect primary tubular epithelial cells with high efficiency [10]. Inoculation of the different tubular cell lines with HTNV at an MOI of 1 revealed enormous differences in the permissiveness despite surface expression of hantaviral entry receptors integrin αvβ3 and CD55 by all three cell lines (Figure 2). HTNV infects and replicates efficiently in HK-2 cells as shown by one third of initially infected cells at day two post infection and about 85% of infected cells on six days post infection (dpi) (Figure 2A,C). In contrast, only about 10% of TH1 and RPTEC/TERT1 cells are susceptible to HTNV infection. In addition, no increase in the number of infected cells was observed over time in these two cell lines. Western blot analysis demonstrates expression of N protein in cellular lysates and cell-free supernatants of all three cell lines (Figure 2B).

### 3.3. Morphological and Functional Consequences in HTNV-Infected HK-2 Cells

**Figure 3:**
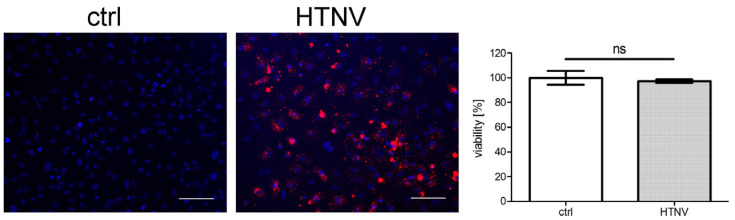
Viability of HTNV-infected HK-2 cells. The viability of HTNV-infected cells was tested at six dpi. Infection was monitored by staining of N protein and more than 95% of cells were infected. Scale bar: 100 µm. The viability of uninfected cells was set to 100%. Three independent experiments were performed. Mean ± SD is shown.

Morphological and functional changes of tubular epithelial cells are observed in renal tissue of patients with HFRS and in hantavirus infection studies with primary human tubular cells in vitro [7,10,18,32]. Therefore, we examined the consequences of HTNV infection in the permissive HK-2 cell line. TH1 and RPTEC/TERT1 cells were excluded from further studies due to their low permissiveness. As observed for primary cells [10], viability of HK-2 cells was not affected by HTNV infection (Figure 3).

**Figure 4:**
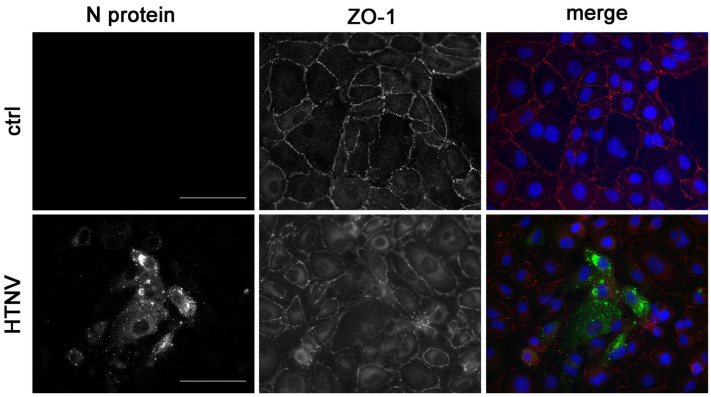
Localization of the tight junction marker protein zonula occludens-1 (ZO-1) in HTNV-infected HK-2 cells. N protein and ZO-1 were stained at three dpi in HTNV-infected and uninfected HK-2 cells. Scale bar: 100 µm.

Cell-to-cell contacts are disturbed in glomerular and tubular cells, as demonstrated by the altered localization of the tight junction marker protein ZO-1 in kidney biopsy samples of HFRS patients and in in vitro infected human glomerular and primary tubular epithelial cells [10]. As shown in Figure 4, disruption of cell-to-cell contacts by redistribution of ZO-1 protein is also observed in infected HK-2 cells. In contrast to the distinct and continuous localization of ZO-1 along the contact sites of neighbored cells in the uninfected control monolayer, the pattern of ZO-1 staining is diffuse in the infected cell population. The ZO-1 protein is redistributed from the cell-to-cell contacts to the cytoplasm.

**Figure 5:**
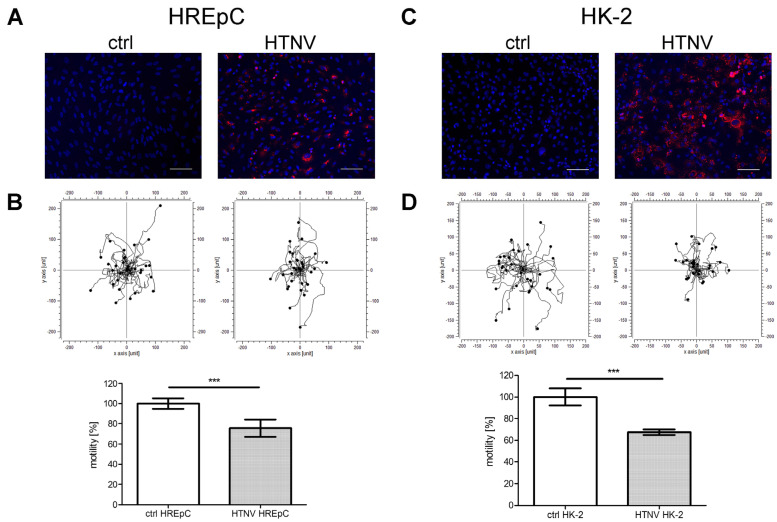
Motility of HTNV-infected human primary epithelial cells (HREpCs) and HK-2 cells. (**A**,**C**) Infection of cells was monitored by immunostaining of N protein. (**B**,**D**) Migration of 30 infected and uninfected cells was analyzed in each experiment. Experiments were performed at six dpi with more than 95% of cells being infected as demonstrated by detection of N protein expression via immunofluorescence. Scale bar: 100 µm. Three independent experiments were performed. Motility of uninfected cells was set to 100%. Shown is mean ± SD. *** *p* < 0.0001.

**Figure 6:**
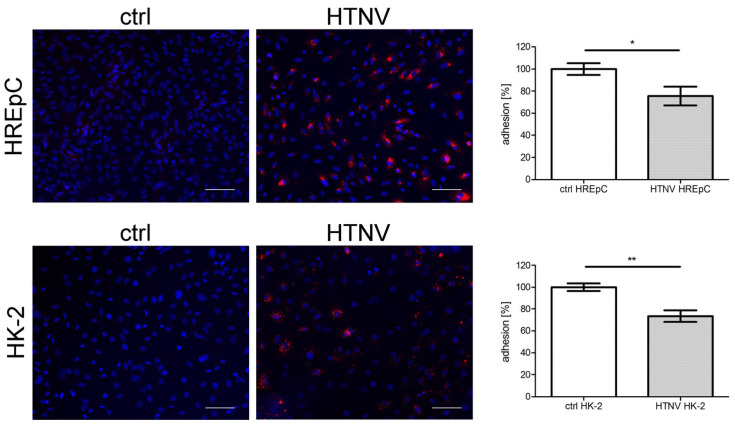
Adhesion of HTNV-infected HREpCs and HK-2 cells. Adhesion of uninfected and infected HREpCs and HK-2 cells was analyzed. Experiments were performed at six dpi with more than 95% of cells being infected as demonstrated by detection of N protein expression by immunofluorescence. Scale bar: 100 µm. Three independent experiments are performed. Adhered uninfected cells were set to 100%. Shown is mean ± SD. * *p* < 0.05; ** *p* < 0.005.

The adhesion and motility capacity of HREpCs infected with PUUV is significantly [18] impaired. Therefore, we compared the impact of HTNV infection on the adhesion and motility capacity of primary tubular epithelial cells and the tubular cell line HK-2 (Figure 5 and Figure 6). Motility was reduced in infected HREpCs (Figure 5A,B) and infected HK-2 cells (Figure 5C,D). The decrease was comparable and not statistically different between primary cells and the cell line. As observed for HTNV-infected HREpCs, the adhesion capacity of infected HK-2 cells was also impaired (Figure 6). The level of reduction of adhesion of infected HK-2 cells does not differ from the impairment observed in infected HREpCs, but it demonstrates a higher statistical significance than in infected HREpCs.

Together, results concerning infection, cell viability, redistribution of ZO-1, impairment of adhesion, and motility in the infected HK-2 cell line correspond closely to the observations made in infected primary tubular epithelial cells. Therefore, HK-2 cells represent an adequate and relevant cell culture model for infection of human tubular cells with HTNV.

### 3.4. Infection of Human Tubular Epithelial Cells with Non-/Low-Pathogenic TULV

**Figure 7:**
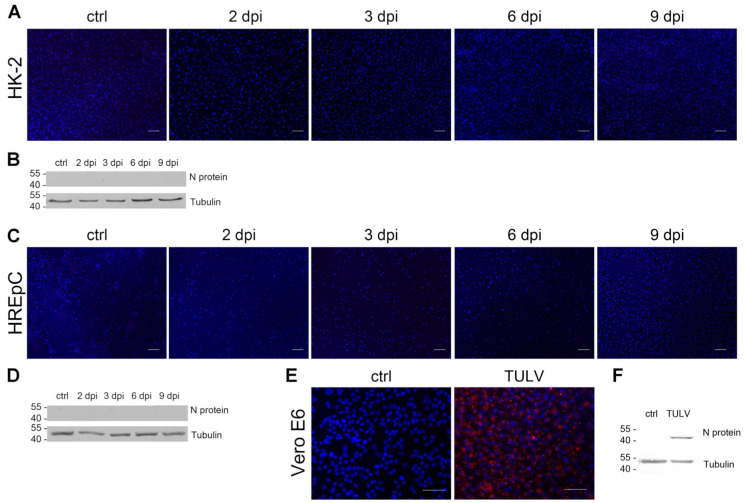
Infection of HK-2 cells and HREpCs with Tula virus (TULV). Cells were inoculated with TULV at an MOI of 1. Cells were analyzed for the expression of N protein by (**A**,**C**) immunofluorescence and (**B**,**D**) Western blot. TULV-infected Vero E6 cells served as positive control for the detection of N protein by (**E**) immunofluorescence staining and (**F**) Western blot analysis. Scale bar: 100 µm.

We showed that analysis of replication kinetics and direct effects of highly pathogenic HTNV in infected HREpCs and HK-2 cells revealed comparable results in primary cells and the cell line. To test the suitability of HK-2 cell line for infection studies with other members of the genus Orthohantavirus, we compared the infection with the non-/low-pathogenic orthohantavirus TULV between primary cells and the cell line (Figure 7A–D). Vero E6 cells infected with TULV served as positive control for the detection of TULV N protein by immunofluorescence and Western blot with anti-PUUV N protein antibodies (Figure 7E,F). Interestingly, neither HK-2 cells (Figure 7A,B) nor HREpCs (Figure 7C,D) were permissive for infection with TULV. We did not detect any cell expressing N protein in the population of primary tubular cells or of the tubular cell line HK-2 (Figure 7A,C). N protein was also not detectable in cellular lysates of HREpCs and of HK-2 cells inoculated with TULV (Figure 7B,D). Together, human tubular epithelial cells are permissive for highly pathogenic HTNV, but not for the infection with non-/low-pathogenic TULV.

## 4. Discussion

The clinical picture of infectious diseases often depends on the specific organ tropism of the infectious agent. The identification of target cells and analysis of direct effects of infection therefore play a key role in the understanding of viral pathogenesis. The clinical picture of HFRS caused by pathogenic Eurasian hantaviruses is characterized by acute kidney injury. Damage of the glomeruli as well as the tubular apparatus is observed in patients with HFRS and contributes to disease severity [7,8,9,10,11]. The identification of kidney cells as target cells of orthohantaviral infection and in vitro studies using human kidney cells provide interesting insights in the pathomechanisms of HFRS. Infection of endothelial and epithelial renal cells results in several cell type-specific morphological and functional alterations that may be directly linked to the clinical picture of HFRS [10,17,20].

The infection of glomerular and tubular cells and the consequences need further investigation and may be facilitated by the use of kidney cell lines. Therefore, we tested three different tubular epithelial cell lines and identified HK-2 cells as a suitable cell culture model. HK-2 cells, in contrast to the other two cell lines (TH1 and RPTEC/TERT1), are permissive to orthohantavirus infection. The strong differences in the permissiveness of the cell lines are difficult to explain. The immortalization of highly specialized cell types is unavoidably associated with a certain level of dedifferentiation affecting morphology and behavior of cells. Such processes may also account for the observed differences in hantaviral infection. The number of infected TH1 and RPTEC/TERT1 cells is low and did not increase. Despite the small amount of infected cells, a substantial level of N protein is detectable in cellular lysates indicating that the amount of N protein does not correlate with the percentage of infected cells. Cell type specific differences in the cellular accumulation of N protein seem to exist and are also observable between different pulmonary cell types [33]. The high levels of intracellular N protein in the absence of viral spread may be the result of cell-type specific antiviral mechanisms or restriction factors, which block the viral replication cycle and particle release leading to the accumulation of viral proteins. N protein is also detected in cell-free supernatants of all three cell lines, but viral spread is only observed in HK-2 cells. Despite the presence of N protein in the supernatant and the surface expression of orthohantaviral receptor integrin αvβ3 and CD55 on all three tubular cell lines, TH1 and RPTEC/TERT1 cells do not support effective replication of HTNV. Absence of viral spread despite the presence of N protein in the supernatant may be due to the release of defective and non-infectious particles [34]. As shown for other cell types, the presence or absence of integrin αvβ3 does not predict the permissiveness of cells: human pulmonary cell types and tubular epithelial cells from bank vole as a hantaviral host species are permissive despite the absence of detectable integrin αvβ3 expression [33,35,36]. Differences in the infection of cells independent of expression or absence of integrin αvβ3 indicate alternative entry routes and receptor usage or the presence of possible cell-type specific restriction factors. Several receptors which mediate hantavirus entry have been described [31,37,38,39,40,41,42,43]. The methods of receptor identification vary enormously and different cell culture systems were used. Blocking with antibodies or ligands, binding assays, recombinant receptor expression in non-permissive cell lines, as well as knock-out of candidate proteins were performed. It would be of interest if one of these receptor candidates is involved in the infection of human renal cell types. The use of HK-2 cells and other renal cell lines with differences in permissiveness may allow the identification of receptor usage or restriction factors involved in orthohantaviral infection.

Besides the analysis of hantaviral entry and viral replication kinetics, in vitro infection studies with a relevant target cell lines are also advantageous for the characterization of hantaviral pathogenesis and virulence. Direct effects in infected cells may contribute to the clinical picture. Analysis of cell-to-cell contacts in kidney biopsy samples of HFRS patients shows the disruption of cell-to-cell contacts in tubules expressing N protein whereas uninfected tubules without N protein expression are not affected and display intact cell-to-cell contacts [10]. As observed in biopsies and in infected primary cells, the localization of the tight junction protein ZO-1 is also disturbed in infected HK-2 cells. In addition to this morphological alteration, infection of HK-2 with HTNV also results in functional consequences by impairment of adhesion and migration capacity. Therefore, the HK-2 cell line represents a suitable and relevant cell culture model with similar replication kinetics together with morphological and functional effects as observed in HTNV-infected primary tubular epithelial cells.

Orthohantaviral infection leads to changes in the transcriptional and proteome profile as shown by in vitro studies in endothelial cells of the human umbilical vein, pulmonary cells, glomerular mesangial cells, and markers of tubular damage are elevated in urinary samples of patients with HFRS [11,44,45]. The analysis and comparison of the transcriptional and proteome profiles of infected HK-2 cells with profiles in urinary samples of patients will help to identify key mediators and signaling cascades involved in pathogenesis of AKI.

The pathogenicity of orthohantaviruses varies enormously between members although they share very high genetic similarity. Differences in cell tropism and replication competence may contribute to the broad range of virulence of hantavirus species. Our results demonstrate that tubular epithelial cells are infected by highly pathogenic HTNV, but are not targeted by TULV, a non-/low-pathogenic member of the genus. This result shows very good accordance with the abortive infection and absence of replication of TULV observed in human mesangial and microvascular endothelial cells of the glomerulus [17,20]. The underlying mechanism of the abortive infection of TULV are not known. Differences in the induction of the immune response are observed in infection with TULV compared to infection with Prospect Hill virus (PHV) or PUUV [17,46]. A detailed analysis of the innate immune response induced by different hantaviruses in kidney cells is necessary to unravel the mechanisms of hantaviral pathogenicity. In addition, differences in the receptor usage of TULV and PUUV may be responsible for the inefficient replication of TULV in tubular cells. TULV is supposed to enter cells via integrin β1 [40,46,47]. Indeed, human tubular epithelial cells express integrin β1 [48,49]. However, the role of integrin β1 in TULV entry was never evaluated. Integrin β1 blocking assays impair the infection with the non-pathogenic hantavirus PHV. However, it remains speculative whether integrin β1 also mediates the entry of TULV. Nevertheless, the inefficient infection of different glomerular cell types and tubular cells may be responsible for the a-/low-pathogenicity of TULV since viral load correlates with disease severity in hantavirus infection [50,51,52].

Further investigations in human renal cells with different virus members are necessary to analyze replication cycle, direct effects of infection, immune modulation, and pathogenicity factors of orthohantaviruses. In addition to the human podocyte cell line CIHP as a glomerular cell type, the use of the tubular epithelial HK-2 cell line will facilitate this future work. Of course, it is to mention that cell culture studies have limitations. In contrast to animal models or organoid models, in vitro infection of different isolated kidney cell types does not mimic the complex situation in the kidney with the interplay of all renal cell types together with effects mediated by the immune system. However, the identification of differences in permissiveness as well as morphological and functional consequences in these in vitro cell culture studies demonstrate the importance of direct effects in the clinical picture of HFRS and emphasizes the usefulness of relevant cell culture models in hantavirus research.

## Data Availability

The data presented in this study are available on request from the corresponding author.
